# Incidence and Risk Factors for Persistent Avascular Retina in Patients with a History of Retinopathy of Prematurity in Mexico

**DOI:** 10.3390/diagnostics16142218

**Published:** 2026-07-16

**Authors:** Marlon García-Roa, César Valdez-Benavides, Kouatzin Aguilar-Morales, Ahmad Santina, Verónica A. Romero-Morales

**Affiliations:** 1 Department of Retina, Instituto Mexicano de Oftalmología, Querétaro 76090, Mexico; drmgroa@gmail.com (M.G.-R.); cvb260897@gmail.com (C.V.-B.); 2 Department of Pediatric Ophthalmology, Instituto Mexicano de Oftalmologia, Querétaro 76090, Mexico; kouatzin.aguilar@imoiap.com.mx; 3 Stein Eye Institute, University of California Los Angeles, Los Angeles, CA 90024, USA; santina.ahmad@gmail.com

**Keywords:** retinopathy of prematurity, persistent avascular retina, anti-VEGF, laser photocoagulation, prematurity

## Abstract

**Background/Objectives**: To determine the incidence of persistent avascular retina (PAR) and identify associated risk factors in patients with a history of retinopathy of prematurity (ROP) evaluated with ultra-widefield imaging. **Methods**: A retrospective case–control study was conducted on patients with a history of ROP evaluated with ultra-widefield imaging at a tertiary care center in Mexico. Clinical variables were analyzed, with PAR as the primary outcome. Associations were assessed using binary logistic regression models. **Results:** Ninety-two patients were included, of whom 12 (13.0%) presented with PAR. Receiving ≥2 anti-VEGF injections was significantly associated with PAR (OR 4.48; 95% CI 1.21–16.64; *p* = 0.032), an association that remained consistent after adjustment for gestational age and maternal factors. Additionally, maternal preeclampsia and chorioamnionitis were independently associated with PAR. **Conclusions**: Persistent avascular retina appears to result from a complex interplay of prenatal and postnatal factors affecting retinal vascular development. Patients requiring repeated anti-VEGF treatment and those exposed to adverse maternal conditions may represent a subgroup at increased risk for incomplete peripheral retinal vascularization, supporting the need for long-term retinal surveillance after ROP regression.

## 1. Introduction

Retinopathy of prematurity (ROP) is a vitreoretinal disorder that affects premature and low birth weight newborns, and it is the leading cause of blindness in childhood worldwide [1]. Retinal vascular development is a tightly regulated process that begins during mid-gestation. Although controversy exists regarding the exact mechanisms in the human infant, retinal vascularization is believed to originate adjacent to the optic nerve via vasculogenesis at 16 weeks’ gestation, in which circulating angioblasts differentiate and form the earliest retinal vascular structures. From these initial vessels, angiogenesis subsequently drives the centrifugal extension of the retinal vasculature toward the ora serrata at 40 weeks’ gestation. This angiogenic process involves endothelial cell proliferation and migration along molecular gradients, as well as the recruitment of mural cells that contribute to the maturation and stabilization of vascular structures. Most retinal vascular development is thought to occur through angiogenesis. Disruption of these tightly coordinated mechanisms in premature infants may result in incomplete vascularization of the peripheral retina, which plays a central role in the pathogenesis of retinopathy of prematurity and may lead to persistent peripheral avascular retina after disease regression [1,2,3]. The pathogenesis of ROP involves a biphasic process. The initial phase is characterized by disruption and arrest of normal retinal vascular development after premature birth with high oxygen levels and low Vascular Endothelial Growth Factor (VEGF) levels, followed by a second phase of hypoxia-driven pathologic neovascularization with high VEGF levels.

The global pooled prevalence of retinopathy of prematurity (ROP) is approximately 31.9% (95% CI 29.0–34.8) among premature infants, with severe ROP affecting 7.5% (95% CI 6.5–8.7) based on a meta-analysis of 139 studies including 121,618 premature infants from 1985–2021. The highest ROP prevalence is found in lower-income countries with high mortality rates [4]. In the United States, the incidence of retinopathy of prematurity (ROP) is estimated at approximately 8.1% among infants considered at risk. Over recent decades, advances in neonatal intensive care, including the use of surfactant therapy and antenatal corticosteroids, have improved survival among extremely premature infants, unintentionally expanding the population vulnerable to ROP [5]. Several systemic and perinatal factors have been associated with the development of retinopathy of prematurity. The most consistently reported risk factors are low gestational age and low birth weight, which reflect the degree of retinal vascular immaturity. Additional factors associated with increased risk include supplemental oxygen exposure, prolonged mechanical ventilation, neonatal sepsis, anemia, blood transfusions, bronchopulmonary dysplasia, intraventricular hemorrhage, and poor postnatal weight gain [6]. Notably, Black and Hispanic infants exhibit significantly higher risk compared with other racial groups [7]. Neonatal sepsis prevalence fluctuates substantially by gestational age and birth weight, with 9–20% of very low birth weight (VLBW) infants developing late-onset sepsis and 0.1–1.9% developing early-onset sepsis. Among infants with ROP, sepsis prevalence is considerably higher, ranging from 13.6–16.7% in large cohorts, reflecting the strong association between sepsis and ROP [8].

Most screening guidelines identify infants at risk for retinopathy of prematurity primarily based on birth weight (BW) and gestational age (GA), which are the main risk factors for disease development. Current recommendations from the American Academy of Pediatrics (AAP), the American Academy of Ophthalmology (AAO), and the American Association for Pediatric Ophthalmology and Strabismus (AAPOS) advise screening all infants with GA ≤ 30 weeks or BW ≤ 1500 g, as well as selected larger infants with an unstable clinical course [9]. In Mexico, approximately 7.7% of live births are premature, placing a large population of infants at risk for ROP. In a screening program conducted in a regional hospital in Querétaro, Mexico, about one-third of screened premature infants developed ROP, including 27.3% with type 1 ROP and 5.3% with type 2 ROP. Given this burden, national screening criteria are broader than those used in many high-income countries, recommending evaluation of infants with gestational age ≤ 34 weeks or birth weight ≤ 1750 g to identify those infants at risk of developing the disease [10]. A standardized classification system has been essential for the diagnosis and management of retinopathy of prematurity. The International Classification of Retinopathy of Prematurity (ICROP), first introduced in 1984, established a uniform framework for describing the disease according to its location (zones), severity (stages), and the presence of vascular activity [11]. Subsequent revisions of the ICROP have been developed through international consensus, incorporating advances in imaging technology and a better understanding of the natural history of the disease [12,13]. Initially, treatment indications for retinopathy of prematurity were based on the concept of threshold disease, which was established in the Cryotherapy for Retinopathy of Prematurity Study (CRYO-ROP). In this landmark study, threshold ROP was defined as stage 3 disease located in zone I or zone II involving at least five contiguous or eight cumulative clock hours of neovascularization in the presence of plus disease [14]. The introduction of the Early Treatment for Retinopathy of Prematurity Study (ETROP) represented a major advance in the management of ROP. According to ETROP recommendations, treatment is indicated for type 1 ROP, defined as zone I with any stage of ROP and “plus disease” (abnormal dilation and tortuosity of retinal blood vessels). Zone I with stage 3 ROP (with or without plus disease). Zone II with stage 2 or 3 ROP accompanied by “plus disease”. These criteria significantly improved visual outcomes by promoting earlier intervention compared with the previous threshold-based treatment strategies [15]. Intravitreal anti-VEGF therapy has emerged as an effective treatment for severe forms of ROP, achieving prompt regression of neovascular activity. Nevertheless, retinal vessels do not progress beyond the level reached by the vascular precursors, leading to a greater persistence of the retinal avascular zone than ablative therapies [1,15,16,17].

Although retinopathy of prematurity is diagnosed in infancy, its consequences may persist into later stages of life. Persistent avascular retina (PAR) has been increasingly recognized as a long-term vascular abnormality in patients with a history of ROP and represents an important structural sequela that may predispose affected individuals to sight-threatening complications such as retinal tears and rhegmatogenous retinal detachment [18]. Studies using ultra-widefield fluorescein angiography have shown that PAR may persist in a substantial proportion of eyes after both spontaneous regression and treatment of ROP [19,20]. In a large cohort study, PAR was observed in approximately one-third of eyes, with a prevalence of 33.3% in eyes with regressed ROP and 31.6% in eyes treated with intravitreal anti-VEGF therapy, and these abnormalities may persist until school age [21,22,23,24]. However, despite growing recognition of this condition, there are currently few studies evaluating the prevalence and risk factors associated with persistent avascular retina in Latin American populations.

## 2. Materials and Methods

This retrospective, observational case–control study was conducted at the Instituto Mexicano de Oftalmología, IAP, a tertiary referral ophthalmologic center in Querétaro, Mexico. The study period extended from January to November 2025. The study adhered to the tenets of the World Medical Association Declaration of Helsinki and was approved by the institutional ethics and research committee of the Instituto Mexicano de Oftalmología. Due to the retrospective nature of the study and the use of anonymized clinical data, the requirement for informed consent was waived.

Medical records of patients with a documented history of Retinopathy of Prematurity (ROP) were systematically reviewed. Patients were identified from the institutional pediatric retina and ROP follow-up databases. Eligible patients included those with either previously treated ROP during infancy or those whose disease had undergone spontaneous regression without intervention.

Most treated patients had received intravitreal anti–vascular endothelial growth factor (anti-VEGF) therapy as primary treatment for type 1 ROP. In our institution, anti-VEGF treatment typically consisted of ranibizumab (Lucentis®; Novartis Pharma AG, Basel, Switzerland) 0.25 mg/0.025 mL, corresponding to approximately half of the standard adult dose, administered as monotherapy according to institutional protocols for severe ROP.

The mean follow-up duration from initial ROP diagnosis or treatment to ultra-widefield imaging evaluation was 35 weeks. Only patients who underwent ultra-widefield retinal imaging during follow-up were considered for inclusion in the present study. Imaging modalities included ultra-widefield fluorescein angiography (UWFA) and/or ultra-widefield color fundus photography, which allowed detailed evaluation of the peripheral retina and the extent of retinal vascularization.

Inclusion criteria were as follows:

• Documented history of ROP diagnosed during infancy
• Availability of at least one ultra-widefield retinal image or angiographic study suitable for evaluation of peripheral retinal vascularization
• Patients older than 65 weeks of postmenstrual age at the time of imaging
• Adequate image quality allowing reliable interpretation of peripheral retinal findings

Exclusion criteria included:

• Presence of other retinal vascular disorders that could affect peripheral vascularization (e.g., familial exudative vitreoretinopathy or retinal vasculitis)
• History of vitreoretinal surgery
• Media opacities or poor-quality images preventing reliable interpretation
• Incomplete medical records lacking relevant perinatal or ophthalmologic data

Ultra-widefield retinal imaging and ultra-widefield fluorescein angiography were performed using the RetCam Envision system (Natus Medical Incorporated, Pleasanton, CA, USA). Pharmacologic pupil dilation was achieved using topical tropicamide 0.8% combined with phenylephrine 5% (T-P®, Laboratorios Sophia S.A. de C.V., Jalisco, Mexico). For angiographic studies, a 10% fluorescein solution (Freedom Ophthalmic Pvt. Ltd., Gujarat, India) was administered intravenously at a dose of 0.1 mL/kg, followed by an isotonic saline flush. Patients were monitored during the procedure for possible systemic reactions. No systemic complications related to fluorescein angiography were observed in this cohort.

All images were independently reviewed by two retina specialists with expertise in pediatric and vitreoretinal diseases (MGR and ANC). Image evaluation focused on the presence and extent of peripheral retinal nonperfusion and vascular abnormalities.

Persistent avascular retina (PAR) was defined as an area of retinal nonperfusion extending two or more optic disc diameters from the ora serrata, exceeding the expected physiological limits of peripheral retinal vascularization and representing incomplete peripheral vascular development following regression of retinopathy of prematurity.

When discrepancies occurred in image interpretation, cases were reviewed jointly, and a final determination was reached by consensus among three retina specialists experienced in the diagnosis and management of ROP.

The primary outcome of the study was the presence of persistent avascular retina (PAR), as determined by ultra-widefield retinal imaging.

Secondary outcomes included evaluation of potential associations between PAR and clinical variables, including:

• Type and number of ROP treatments
• Perinatal characteristics
• Maternal and neonatal risk factors

Categorical variables were summarized using frequencies and percentages, whereas continuous variables were expressed as means with standard deviations or medians with interquartile ranges, depending on data distribution.

To evaluate potential independent associations with persistent avascular retina (PAR) while minimizing the risk of model overfitting, focused binary logistic regression models were constructed using SPSS v26. The analysis included 92 patients, of whom 12 developed PAR. Given the limited number of outcome events, larger multivariable models including multiple simultaneous covariates were avoided. Instead, a series of parsimonious two-predictor logistic regression models was performed, each including the primary therapeutic variable of interest, defined as treatment intensity with ≥2 anti-VEGF injections, together with one clinically relevant covariate.

This strategy was selected to provide adjusted estimates while preserving statistical stability as much as possible in the context of a relatively small number of PAR events. Variables were selected based on clinical relevance and/or findings from the univariate baseline analysis. The resulting adjusted odds ratios should therefore be interpreted as focused exploratory models rather than as a definitive comprehensive multivariable prediction model.

## 3. Results

Between 1 January 2025 and 30 November 2025, a total of 122 newborns were diagnosed with retinopathy of prematurity (ROP) at our institution. Of these, 92 patients met the inclusion criteria and were included in the present analysis. The study population consisted of 56 males (60.9%) and 36 females (39.1%). Eighty patients (87.0%) were delivered by cesarean section.

**Figure 1:**
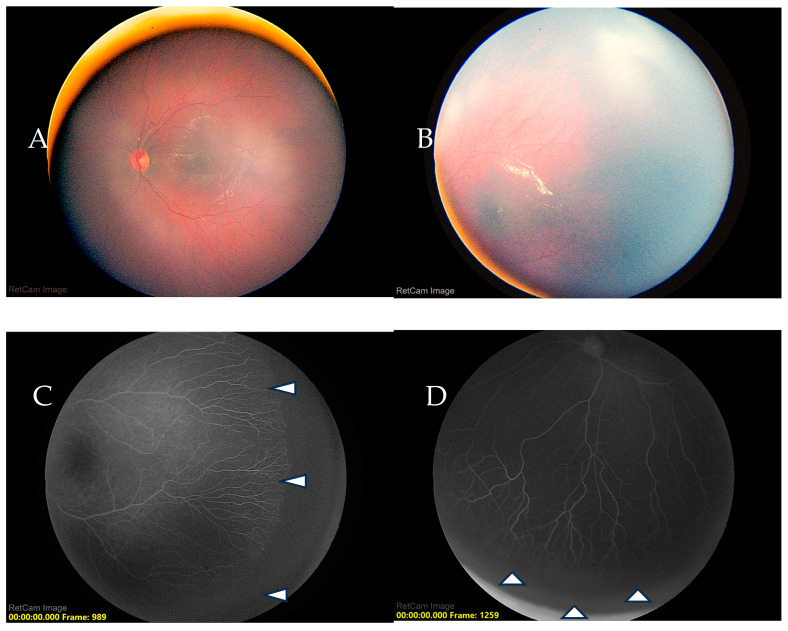
Multimodal retinal imaging of a patient with a history of retinopathy of prematurity (ROP). The patient was born at 31 weeks of gestational age with a birth weight of 1645 g and a history of type 1 ROP. (**A**) Color fundus photograph of the posterior pole. (**B**) Color fundus image of the temporal retina. (**C**) Ultra-widefield fluorescein angiography demonstrating peripheral avascular areas in the temporal retina. (**D**) Ultra-widefield fluorescein angiography showing inferior peripheral avascular zones (indicated by white arrowheads).

The mean gestational age at birth was 30.7 ± 2.6 weeks, and the mean birth weight was 1400.2 ± 455.9 g. Persistent avascular retina (PAR) was identified in 12 patients (13.0%), of whom 6 (50%) were male (Figure 1).

Among patients diagnosed with PAR, 9 (75%) had previously received intravitreal anti-VEGF therapy, whereas 3 patients (25%) had a history of spontaneous regression of ROP without treatment. Patients with PAR tended to have lower gestational age and lower birth weight compared with those without PAR. Specifically, the mean gestational age was 29.5 ± 2.51 weeks in the PAR group versus 30.9 ± 2.56 weeks in patients without PAR, and the mean birth weight was 1269.16 ± 452.58 g versus 1419.88 ± 455.87 g, respectively.

Regarding treatment characteristics, 9 patients (75%) with PAR had received at least one intravitreal anti-VEGF injection, and 5 patients (41.7%) had received two or more doses of anti-VEGF therapy. The mean interval between the first and last anti-VEGF injection was 50 ± 11.3 days (range, 40–69 days). Additionally, the mean time from anti-VEGF therapy to subsequent laser photocoagulation was 226.8 ± 58.9 days (range, 140–353 days) (Table 1).

After adjustment for gestational age, treatment with ≥2 anti-VEGF injections showed a positive association with PAR, with an adjusted odds ratio suggesting approximately a four-fold increase in the odds of PAR compared with patients receiving fewer than two injections or no injections. This association approached, but did not reach, conventional statistical significance thresholds (aOR = 3.744; 95% CI: 0.973–14.408; *p* = 0.055).

Gestational age showed a protective direction of association, with each additional week of gestation associated with lower odds of PAR. However, this association was not statistically significant in the adjusted model (aOR = 0.819; 95% CI: 0.644–1.079; *p* = 0.132).

Model 2 evaluated treatment intensity after adjustment for maternal preeclampsia. Maternal preeclampsia was independently associated with increased odds of persistent avascular retina (aOR 6.38; 95% CI 1.51–26.94; *p* = 0.012). Treatment with ≥2 anti-VEGF injections remained positively associated with PAR; however, the association did not reach statistical significance after adjustment for preeclampsia (aOR 3.70; 95% CI 0.91–15.05; *p* = 0.068).

Model 3 assessed the association between treatment intensity and persistent avascular retina after adjustment for maternal chorioamnionitis. The overall model was statistically significant (Omnibus Test of Model Coefficients, *p* < 0.05). Treatment with ≥2 anti-VEGF injections remained independently associated with PAR, conferring an approximately six-fold increase in the odds of PAR (aOR 6.00; 95% CI 1.44–24.20; *p* = 0.012). Maternal chorioamnionitis was also independently associated with PAR (aOR 8.80; 95% CI 1.18–65.48; *p* = 0.034) (Table 2).

Laser photocoagulation was not included as an independent baseline predictor in the logistic regression models. In this cohort, all 12 patients with PAR underwent subsequent laser photocoagulation, whereas none of the 80 patients without PAR did. This distribution resulted in complete data separation, yielding an infinite unadjusted odds ratio and precluding stable estimation in conventional logistic regression models.

More importantly, laser photocoagulation represented a subsequent therapeutic intervention performed in response to persistent peripheral avascularity, rather than a pre-existing baseline exposure. Therefore, laser should not be interpreted as an independent risk factor for PAR. Instead, it should be considered a clinical management marker or consequence of PAR identification during follow-up.

## 4. Discussion

**Figure 2:**
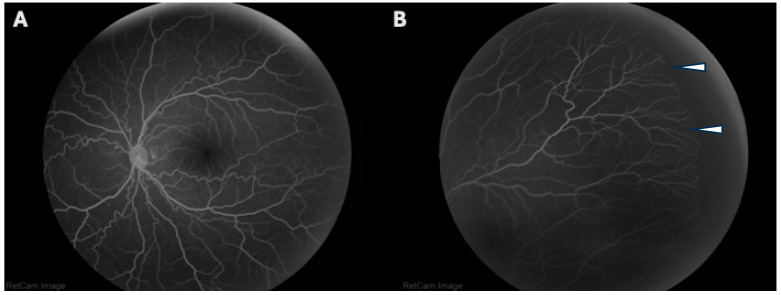
Ultra-Wide Field Fluorescein angiography shows no evidence of persistent avascular retina in the posterior pole (**A**), while temporal peripheral imaging demonstrates persistent avascular retina (**B**) (indicated by white arrowheads).

In this retrospective case–control study, we found that persistent avascular retina (PAR) was present in 13.0% of patients with a history of retinopathy of prematurity (ROP) in our cohort (Figure 2). The prevalence of PAR observed in our study appears lower than that reported in several studies using ultra-widefield fluorescein angiography, where persistent peripheral avascularity has been identified in approximately one-third of eyes with a history of ROP [24]. The lower PAR prevalence in our study compared with prior reports may reflect differences in study populations and methodology. Unlike studies focused predominantly on severe or anti-VEGF–treated ROP, our cohort included both treated and spontaneously regressed cases, potentially reducing the overall burden of persistent avascularity. Differences in imaging timing, follow-up duration, ultra-widefield angiography protocols, and PAR diagnostic criteria may also explain the observed discrepancy. In our study population, the men/women ratio with ROP was 60.9/39.1, which is consistent with approximately 55:45 (or 1.22:1) ratio reported in the other studies [25]. However, no specific data exists on the sex distribution for persistent avascular retina (PAR) in ROP patients.

In our study, persistent avascular retina appeared to be associated with both postnatal treatment intensity and selected prenatal maternal factors. Focused binary logistic regression models showed that treatment with ≥2 anti-VEGF injections had a consistent positive direction of association with PAR across all adjusted models. Although this association did not reach conventional statistical significance in the models adjusted for gestational age and maternal preeclampsia, the magnitude and direction of the adjusted odds ratios were consistent. In the model adjusted for maternal chorioamnionitis, treatment with ≥2 anti-VEGF injections was significantly associated with increased odds of PAR.

These findings support the hypothesis that repeated anti-VEGF exposure may contribute to delayed or incomplete peripheral retinal vascularization in some patients. This interpretation is biologically plausible, as VEGF is involved not only in pathological neovascularization but also in physiological retinal vascular development. Therefore, repeated pharmacologic suppression of VEGF signaling could theoretically interfere with continued centrifugal vascular growth toward the retinal periphery after regression of active retinopathy of prematurity.

However, this association should not be interpreted as definitive evidence of causality. The observational nature of the study, the limited number of PAR events, and the wide confidence intervals indicate that the magnitude of the effect remains imprecise. Furthermore, treatment intensity may also reflect greater baseline disease severity, because infants requiring repeated anti-VEGF injections may have had more aggressive or persistent ROP phenotypes. For this reason, the observed association between ≥2 anti-VEGF injections and PAR should be considered hypothesis-generating and should be validated in larger cohorts with more detailed adjustment for baseline ROP severity, zone, stage, plus disease, birth weight, oxygen exposure, and follow-up duration.

Consistent with our observations, Perente et al. [26] reported persistent avascular retina in a substantial proportion of patients treated with anti-VEGF therapy, with 29.3% of eyes failing to reach zone III vascularization at a mean post-menstrual age of 95.09 ± 3.80 weeks, reinforcing the concept that anti-VEGF treatment may not guarantee complete physiologic peripheral retinal vascularization. Meng et al. [27] reported 29.8% PAR, with nearly one quarter demonstrating retinal vascularization limited to zone 2, and the most important risk factors in this group of patients were lower birth weight and more aggressive forms of ROP. The re-treatment rate was 36.3% compared with 41.6% in our study.

The associations observed with maternal preeclampsia and chorioamnionitis suggest that prenatal vascular and inflammatory conditions may also contribute to the development of persistent peripheral avascularity. Preeclampsia is characterized by maternal endothelial dysfunction and altered angiogenic balance, which may influence fetal vascular development. Similarly, chorioamnionitis reflects an intrauterine inflammatory environment that may affect fetal endothelial function and postnatal vascular maturation. In this context, PAR may not represent only a postnatal treatment-related finding, but rather the final expression of multiple prenatal and postnatal influences affecting retinal vascular development.

The significant association between chorioamnionitis and PAR in the focused adjusted model is particularly interesting. When chorioamnionitis and treatment intensity were included together, both variables were significantly associated with PAR. This may suggest a clinically relevant relationship between prenatal inflammatory exposure and the effect of repeated anti-VEGF therapy on peripheral vascular maturation. Nevertheless, because no formal interaction term was tested and because of the limited number of PAR events, this result should be interpreted cautiously. Future studies with larger sample sizes are needed to determine whether prenatal inflammation modifies the relationship between anti-VEGF treatment intensity and persistent avascular retina.

Laser photocoagulation showed complete alignment with PAR in this cohort, as all patients with PAR underwent subsequent laser treatment and no patients without PAR required laser. This finding emphasizes the clinical importance of persistent peripheral nonperfusion during long-term surveillance after ROP treatment. However, laser should not be interpreted as a risk factor for PAR, since it was performed after identification of persistent avascular retina. Rather, it represents a clinical consequence of PAR detection and a marker of management decisions in patients with persistent peripheral avascularity.

Overall, these findings suggest that PAR may arise from a multifactorial pathway involving retinal immaturity, adverse prenatal vascular or inflammatory exposures, baseline ROP severity, and postnatal treatment intensity. The consistent direction of association between repeated anti-VEGF injections and PAR supports the need for careful long-term peripheral retinal evaluation after anti-VEGF therapy, especially in infants requiring multiple injections or those with relevant maternal risk factors. Nonetheless, these results should be interpreted with caution due to the exploratory nature of the adjusted models, the small number of outcome events, and the imprecision reflected by the wide confidence intervals.

Our findings are consistent with the growing body of literature highlighting persistent avascular retina (PAR) as a frequent long-term vascular sequela in patients with a history of retinopathy of prematurity. Tan et al. reported that PAR may occur not only in eyes treated with intravitreal anti-VEGF therapy but also in eyes that undergo spontaneous regression of ROP, suggesting that incomplete peripheral vascularization may be related to the underlying pathophysiology of the disease rather than treatment alone. In their cohort, persistent areas of peripheral avascular retina were identified during follow-up examinations using fluorescein angiography, emphasizing the importance of advanced imaging techniques in detecting these abnormalities [23].

The development of persistent avascular retina in patients with a history of ROP is likely driven by a combination of systemic and local factors. Extreme prematurity and low birth weight reflect immaturity of the retinal vasculature at birth, predisposing to incomplete peripheral vascularization. Anti-VEGF therapy, while effective in controlling acute neovascular activity, may suppress physiologic VEGF signaling necessary for normal vascular growth, resulting in arrested peripheral vascular extension. Additionally, systemic insults such as neonatal sepsis, respiratory distress requiring surfactant, and prolonged oxygen exposure may contribute to endothelial dysfunction and altered angiogenic signaling, further impairing peripheral retinal vascular maturation.

Late retinal complications have also been reported in patients with a history of untreated retinopathy of prematurity. In a large multicenter case series including 363 eyes from 186 patients, Hamad et al. [21] described a wide spectrum of late peripheral retinal abnormalities associated with persistent avascular retina. The most common findings included lattice-like degeneration (54.0%), atrophic retinal holes (34.7%), retinal tears (30.6%), and retinal detachments (38.6%). Additionally, incomplete peripheral retinal vascularization was observed in a significant proportion of eyes, suggesting that persistent avascular retina may contribute to long-term vitreoretinal interface abnormalities and peripheral retinal pathology. These findings highlight the importance of long-term surveillance in patients with a history of ROP, even in eyes that did not require treatment during infancy.

Finally, data regarding the prevalence and risk factors for persistent avascular retina in Latin American populations remain extremely limited. Most published studies evaluating PAR have been conducted in North American, European, or Asian cohorts [19,23,28,29,30]. Given the relatively high incidence of premature birth and retinopathy of prematurity in middle-income countries, further research is needed to better characterize the long-term vascular outcomes of these patients in different geographic and healthcare settings. Regional differences in neonatal care practices, screening strategies, and treatment approaches may influence the prevalence and clinical consequences of persistent avascular retina.

The findings of this study have important clinical implications for the long-term management of patients with a history of retinopathy of prematurity. Persistent avascular retina represents a structural marker of incomplete peripheral retinal vascularization that may remain undetected during routine fundus examination. The increasing availability of ultra-widefield imaging and fluorescein angiography has improved the ability to identify these peripheral abnormalities and may facilitate earlier recognition of eyes at risk for late vitreoretinal complications. Previous studies have suggested that areas of persistent peripheral nonperfusion may predispose patients to lattice degeneration, retinal breaks, and rhegmatogenous retinal detachment later in life, as well as neovascularization zones in avascular retina [21]. Therefore, patients with a history of ROP, particularly those born at lower gestational ages or treated with anti-VEGF therapy, may benefit from laser ablative treatment at avascular zones or periodic long-term retinal evaluation. Early identification of peripheral vascular abnormalities could allow timely intervention and may help reduce the risk of vision-threatening complications associated with persistent avascular retina such as retinal detachment or vitreous hemorrhage.

This study has several limitations. First, the number of PAR events was limited, with only 12 affected patients in the analyzed cohort. This restricted the number of covariates that could be included in the regression models and prevented construction of a larger fully adjusted multivariable model. Second, the confidence intervals around several estimates were wide, reflecting imprecision in the estimated magnitude of association. Third, the observational design does not allow causal inference. Fourth, treatment with ≥2 anti-VEGF injections may be a marker of more severe or persistent baseline ROP rather than an isolated causal factor. The laser photocoagulation could not be evaluated as a baseline predictor because it was performed after PAR recognition and demonstrated complete data separation. Finally, it has been well established in several studies that low birth and low gestational age are major risk factors for the development of ROP, but in our cohort 87% of the patients were delivered via cesarean, so gestational age could be influenced by the clinician.

Despite these limitations, the study provides clinically relevant exploratory evidence suggesting that persistent avascular retina may be associated with both repeated anti-VEGF exposure and prenatal maternal factors such as preeclampsia and chorioamnionitis. These findings support the need for larger prospective studies to better define risk factors for PAR and to establish optimal long-term follow-up strategies after anti-VEGF treatment for retinopathy of prematurity.

Additionally, fluorescein angiography was not performed at standardized ages, which may have influenced the detection of PAR. Differences in reported prevalence may be related to variations in study design, patient populations, treatment strategies, imaging modalities, and the timing of angiographic evaluation. Studies that perform fluorescein angiography during early childhood or school age tend to report higher rates of persistent peripheral nonperfusion, suggesting that PAR detection may depend on long-term follow-up and the use of advanced widefield imaging technologies. Despite these limitations, our study provides important insights into the prevalence and clinical characteristics of persistent avascular retina in a Mexican cohort of patients with a history of retinopathy of prematurity. To our knowledge, relatively few studies have evaluated this condition in Latin American populations. Our findings contribute to the growing recognition that persistent peripheral retinal vascular abnormalities may remain present long after the regression of acute ROP.

## Figures and Tables

**Table 1:** Baseline demographic and clinical characteristics.

Variable	PAR (*n* = 12)	No PAR (n = 80)	*p*-Value
Gestational age (weeks), mean ± SD	29.5 ± 2.51	30.9 ± 2.56	0.093
Birth weight (g), mean ± SD	1269.16 ± 452.58	1419.88 ± 455.87	0.300
Cesarean delivery, n (%)	11 (91.7%)	69 (86.3%)	1.000
1 anti-VEGF injection, n (%)	9 (75%)	48 (60%)	0.363
≥2 anti-VEGF injections, n (%)	5 (41.7%)	11 (13.8%)	0.032
Male sex, n (%)	6 (50%)	50 (62.5%)	0.528

**Table 2:** Focused Binary Logistic Regression Models for Factors Associated with Persistent Avascular Retina.

Model/Independent Variables	Adjusted Odds Ratio	95% Confidence Interval	*p*-Value	Model Fit
**Model 1: Adjustment for Gestational Age**				χ^2^ = 8.17, *p* = 0.017
≥2 anti-VEGF injections	3.744	0.973–14.408	0.055	
Gestational age, per week	0.819	0.644–1.079	0.132	
**Model 2: Adjustment for Maternal Preeclampsia**				*p* < 0.05
≥2 anti-VEGF injections	3.699	0.909–15.051	0.068	
Maternal preeclampsia	6.383	1.514–26.935	0.012 *	
**Model 3: Adjustment for Maternal Chorioamnionitis**				*p* < 0.05
≥2 anti-VEGF injections	6.000	1.488–24.199	0.012 *	
Maternal chorioamnionitis	8.800	1.183–65.475	0.034 *	

## Data Availability

The data presented in this study are available on request from the corresponding author. The data are not publicly available due to privacy and ethical restrictions.

## Abbreviations

The following abbreviations are used in this manuscript:

ROP	Retinopathy of prematurity
PAR	Persistent avascular retina
VEGF	Vascular Endothelial Growth Factor
UWFA	Ultra-Wide Field Angiography
